# Developing Technology to Mobilize Personal Strengths in People with Chronic Illness: Positive Codesign Approach

**DOI:** 10.2196/10774

**Published:** 2018-06-05

**Authors:** Jelena Mirkovic, Stian Jessen, Olöf Birna Kristjansdottir, Tonje Krogseth, Absera Teshome Koricho, Cornelia M Ruland

**Affiliations:** ^1^ Center for Shared Decision Making and Collaborative Care Research Division of Medicine Oslo University Hostipal Oslo Norway; ^2^ Faculty of Medicine University of Oslo Oslo Norway; ^3^ Center for Learning and Mastery for Children Division of Paediatric and Adolescent Medicine Oslo University Hospital Oslo Norway

**Keywords:** patient personal strengths, participatory design, codesign, appreciative inquiry, service design, positive computing, positive technology, chronic disease, eHealth, mHealth, patient requirements, patient participation

## Abstract

**Background:**

Emerging research from psychology and the bio-behavioral sciences recognizes the importance of supporting patients to mobilize their personal strengths to live well with chronic illness. Positive technology and positive computing could be used as underlying design approaches to guide design and development of new technology-based interventions for this user group that support mobilizing their personal strengths.

**Objective:**

A codesigning workshop was organized with the aim to explore user requirements and ideas for how technology can be used to help people with chronic illness activate their personal strengths in managing their everyday challenges.

**Methods:**

Thirty-five participants from diverse backgrounds (patients, health care providers, designers, software developers, and researchers) participated. The workshop combined principles of (1) participatory and service design to enable meaningful participation and collaboration of different stakeholders and (2) an appreciative inquiry methodology to shift participants’ attention to positive traits, values, and aspects that are meaningful and life-giving and stimulate participants’ creativity, engagement, and collaboration. Utilizing these principles, participants were engaged in group activities to develop ideas for strengths-supportive tools. Each group consisted of 3-8 participants with different backgrounds. All group work was analysed using thematic analyses.

**Results:**

Participants were highly engaged in all activities and reported a wide variety of requirements and ideas, including more than 150 personal strength examples, more than 100 everyday challenges that could be addressed by using personal strengths, and a wide range of functionality requirements (eg, social support, strength awareness and reflection, and coping strategies). 6 concepts for strength-supportive tools were created. These included the following: a mobile app to support a person to store, reflect on, and mobilize one’s strengths (Strengths treasure chest app); “empathy glasses” enabling a person to see a situation from another person’s perspective (Empathy Simulator); and a mobile app allowing a person to receive supportive messages from close people in a safe user-controlled environment (Cheering squad app). Suggested design elements for making the tools engaging included: metaphors (eg, trees, treasure island), visualization techniques (eg, dashboards, color coding), and multimedia (eg, graphics). Maintaining a positive focus throughout the tool was an important requirement, especially for feedback and framing of content.

**Conclusions:**

Combining participatory, service design, and appreciative inquiry methods were highly useful to engage participants in creating innovative ideas. Building on peoples’ core values and positive experiences empowered the participants to expand their horizons from addressing problems and symptoms, which is a very common approach in health care today, to focusing on their capacities and that which is possible, despite their chronic illness. The ideas and user requirements, combined with insights from relevant theories (eg, positive technology, self-management) and evidence from the related literature, are critical to guide the development of future more personalized and strengths-focused self-management tools.

## Introduction

### The Importance of Personal Strengths for Illness Self-Management

Living with chronic illness is highly demanding. It often requires a person to simultaneously manage multiple symptoms, disability, complex medical regimens, difficult lifestyle adjustments, and emotional consequences (eg, depression, fear, and anxiety) [1,2]. Learning how to manage these illness-related challenges is a crucial part of self-management. Self-management is defined as “the tasks that individuals must undertake to live well with one or more chronic conditions” [3]. To support patients in performing these tasks, a range of technology-based self-management programs and interventions exist, which focus primarily on supporting patients in learning, developing, and practicing new skills and knowledge required to manage their symptoms and problems as well as live healthy and satisfying lives [4,5]. However, so far, technology-assisted self-management interventions are usually designed to support problem-solving approaches to self-management, for example, monitoring and providing information on how to manage symptoms and biological outcomes.

Increasing evidence from psychology and the bio-behavioral sciences suggests that the focus on pathology and health deficits that has considerably dominated the health care discourse may not be optimal to help patients reach their best health potential. For example, sustained attention on symptoms and problems can create a downward spiral of sensitization with negative emotional and moral implications (eg, fear, anger, and injustice) as well as serve as a constant reminder of negative aspects of the persons’ illness [6,7]. On the other hand, shifting the focus to personal strengths and resources may counteract these processes as it creates a horizon of possibilities accompanied by a sense of control and mastery, which inspire mobilization and positive action [8]. While a focus on strengths does not ignore the patients’ problems, it shifts the attention from peoples’ deficits to addressing their health issues in light of their individual capacities, talents, competencies, possibilities, and values [9]. Surprisingly, the crucial role of patients’ personal strengths has not been directly addressed in the self-management literature and very few interventions have been designed that specifically support patients in identifying and mobilizing their personal strengths in the self-management of their illness.

### Positive Psychology and Personal Strengths

A concept of character strengths originated in positive psychology and has been defined as “the characteristics people use to achieve well-being and to flourish, and include attributes such as hope, gratitude, love of learning, honesty, and humor” [10]. In addition to its emphasis on utilizing personal strengths, positive psychology is used as a more general term for the study of positive emotions, positive character traits, and institutions that enable a person to flourish (eg, families and communities) [11]. In relation to health and chronic illness management, the term “patient strengths” (sometimes also referred as health assets) is very often used as a much wider concept that covers internal strengths (eg, optimism, sense of meaning in life, acceptance, and positive emotions), external strength qualities (eg, supportive family, neighborhood and institutions, and stable socioeconomic status), and mastering and coping strategies (eg, meaningful priorities, changing perspective, and being vigilant) that patients use to meet their day-to-day health-related challenges [12-14].

Using strengths have been found to broaden people’s thought-action repertoires, to encourage them to discover novel lines of thinking and behavior, and to increase intellectual, social, and psychological resources [15,16]. Some studies have shown the potential of strengths-based interventions to positively influence healthier lifestyle practices [17], increase mood and happiness [11], promote the efficacy of health management activities [14], and improve general health and well-being [18]. Additionally, the literature reports numerous studies that use technology to deliver positive psychology interventions to wider user groups, both to help them identify and raise awareness and build on, and use more of, their personal strengths in everyday life [11,19,20]. Strengths-based interventions are more commonly evaluated and used in the fields of positive psychology [10], social work [21], community development [22], and business [23]; however, such interventions have been largely unexplored in the field of health care and more specifically, for people with chronic illness.

### Positive Technology and Positive Computing

Positive technology and positive computing approaches to the design of technology introduce underlying design principles and guidelines that are highly relevant for developing strength-supportive interventions. Sander’s term, positive computing, was introduced as “the study and development of information and communication technology that is consciously designed to support people's psychological flourishing in a way that honors individuals’ and communities’ different ideas about the good life” [24]. Positive computing refers to the design of technology that helps people to be “who they want to be” and supports them to better address negative situations and challenges in life [24]. This concept has been further expanded by Calvo and Peters, who in their recent book explore the potential of technology to positively influence different areas of peoples’ lives, such as human positive functioning, positive emotions, motivation, engagement and flow, mindfulness, and empathy [25].

The positive computing concepts were further explored by Riva and colleagues with a specific focus on improving the quality of peoples’ personal experiences [26,27]. They introduced the term positive technology, a scientific and applied approach to “use technology to enhance the features of our personal experiences with the goal of increasing wellness, and generating strengths and resilience in individuals, organizations and society” [26]. Positive technology is classified based on its effects on a specific feature of our personal experience: (1) hedonic: technologies used to induce positive and pleasant experiences, (2) eudemonic: technologies used to support individuals in engaging and self-actualizing experiences, and (3) social or interpersonal: technologies used to support and improve the connection between individuals, groups, and organizations. The authors argue that by positively influencing the positive and self-actualizing experiences and improving interpersonal connections, the positive technologies have the potential to increase people's engagement in self-management activities and their role in the partnership with health care providers [26]. While research has explored the role of positive technology and computing approaches in the general population (eg, [28-30]), the potential of applying positive technology principles in chronic illness management is still in early stages and needs to be further explored.

Successful development of electronic interventions that build on the principles of positive computing and positive technology require multidisciplinary partnerships that explore the design and shape of digital experiences to support human flourishing [25]. This goes beyond mere user acceptance of the system and requires the technology to also be enjoyable, exciting, engaging, and suitable for users’ needs [24,31,32]. The potentials of technology can only be fully exploited when end users and other stakeholders are involved in the design process and their needs and the specifics of the context (both organizational and that of the individual user) in which the technology will be used are taken into consideration. Therefore, close collaboration between different stakeholders, multidisciplinary research teams, and system designers and developers from the early stage of development is a main requirement in the development of successful system design and implementation processes [31].

The study presented in this article is a part of a larger project funded by the Research Council of Norway titled “The Power of Personal Strengths – using gamification to support patients in chronic illness management”. The goal of the project is to build on the concepts and principles of positive computing and positive technology design to develop and evaluate a gamified application that helps people with chronic illnesses identify and mobilize their personal strengths in illness self-management. In this paper, we describe the applied methods and results of a whole-day codesign workshop with a range of stakeholders (patients, health care providers, designers, software developers, and researchers) organized as part of the project. The main goal of the workshop was to identify and collaboratively explore stakeholders’ requirements and cocreate ideas for a technology-supported self-management tool that integrates and builds on patients’ strengths.

## Methods

### Design Approach

The overall design approach in the whole research project combines participatory design and service design methodologies. Participatory design is a design methodology that promotes close collaboration, common understanding, and mutual learning between designers and users in designing a product [33]. Service design also relies heavily on collaboration but further focuses on developing entire services that support value cocreation between customers and service organizations [34]. As such, it typically includes a wider group of stakeholders that are relevant to and involved with the service, not only end users and designers. Therefore, the goal of applying this integrated design approach for our research project was to promote involvement of wide group of stakeholders throughout the development process, and also to give them an important and meaningful role in the design and decision-making processes.

The codesign workshop described in this study was organized in a similar way to what is often termed a Future Workshop within participatory design methodology [35]. Such workshops are often used for developing new and innovative ideas and contain three phases: (1) The critique phase where one openly discusses and presents issues surrounding the topic; (2) The fantasy phase where one creates and suggests possible or impossible solutions or ideas about solutions for the issue(s), and finally (3) The concretization phase where one tries to shape the proposed ideas into something concrete and realizable [35]. However, in our approach, instead of critiquing current systems and focusing on problem-solving, we applied basic concepts of appreciative inquiry methodology to guide the organization of workshop tasks and frame the questions in all three phases with a positive stance [36]. An appreciative inquiry methodology approach aims to focus peoples’ attention on positive traits and values, and discovery of “what gives life” to a system when it is most effective and most constructively capable, and how this state can be extended and improved [36]. Through this process, the goal is not only to explore the system’s capacity to apprehend, anticipate, and heighten positive potential but also to evoke positive emotions with participants during this process. Previous research has shown that when people experience positive emotions, their attention span lengthens, they are curious, and they simultaneously hold multiple perspectives [37]. Facilitating the participants’ curiosity and positivity in this manner can encourage them to be more open to viewing things from different perspectives, and this is more likely to trigger insights and inspire innovation and, as a result, open up new possibilities for action [36]. The philosophy of appreciative inquiry further argues that inquiry and change are deeply interconnected. The type and form of the questions will not only guide the conversation and cooperation between participants but also, at the same time, it will shape what people discover and pursue and instill certain images and expectations of their future, which will further attract energy and mobilize intention and action. Thus, the rationale for using the appreciative inquiry approach was twofold: (1) to engage users in more positively oriented, creative cocreation activities and facilitate a richness of creative ideas; and (2) to engage and empower users to reflect on their situation from new perspectives and look for new possibilities for capacity building and flourishing that is beyond the problem-focused thinking that is predominant in health care today.

Often in the literature, methods for involving stakeholders are only superficially described, leaving other researchers without the necessary detail to reapply the methods. As we applied a novel way to design a codesign workshop, the methods are purposely described in more detail in the following section.

### Participants

In total, 35 participants (13 male, 22 female) from diverse backgrounds took part in the workshop (Table 1). Recruitment was done through patient organizations such as the Norwegian competence center for self-help and the youth council of a local hospital (8/35, 23%), center for family and professional carers (2/35, 6%), the professional network of the project team (20/35, 57%), and by inviting participants from earlier strengths-related studies (5/35, 14%). The study was approved by the Privacy Protection Committee at Oslo University Hospital and all participants signed an informed consent.

### Workshop Procedure

#### Introduction

The workshop started with a short welcome and introduction of the project, after which the workshops’ goals and rules were presented. The rules were formulated to promote joint work and collaboration between participants (eg, listen actively to others, build on each other's ideas, show curiosity about others in the group and their ideas). To set the stage and energize the participants, a patient representative, also a participant from one our previous research studies, shared his personal story about how he uses his own strengths to manage daily challenges when living with chronic illness, or chronic pain in this specific case. This was followed by a group exercise where participants were asked to characterize and reflect on the personal strengths of other people—in our case we used the patient representative who had shared his story and another fictional character from a movie. The purpose of this exercise was to engage the participants in a shared emotional experience, help them think about personal strengths, and prepare them for noticing the strengths of others or themselves in the future [38]. Additionally, this also served as a powerful example they could to draw upon later during other activities.

#### Working in Small Groups

The participants were then divided into 6 smaller groups, aiming to distribute participants with different backgrounds evenly within the groups. Each group consisted of 3-8 participants. Table 1 shows the distribution of participants across groups.

Each group had one facilitator and one observer. The main role of the facilitator was to facilitate the activities and support the participants during this process, while the observer was in charge of taking notes and provided additional support for the facilitator when needed. All facilitators were researchers (4/6, 67%) or research assistants (2/6, 33%) from the project. Observers were other researches (2/6, 33%) or part of the staff (4/6, 67%) from the research center. Both facilitators and observers gained detailed group training and guidance about the activities, tasks, and their role during the workshop.

#### Activity 1

**Figure 1:**
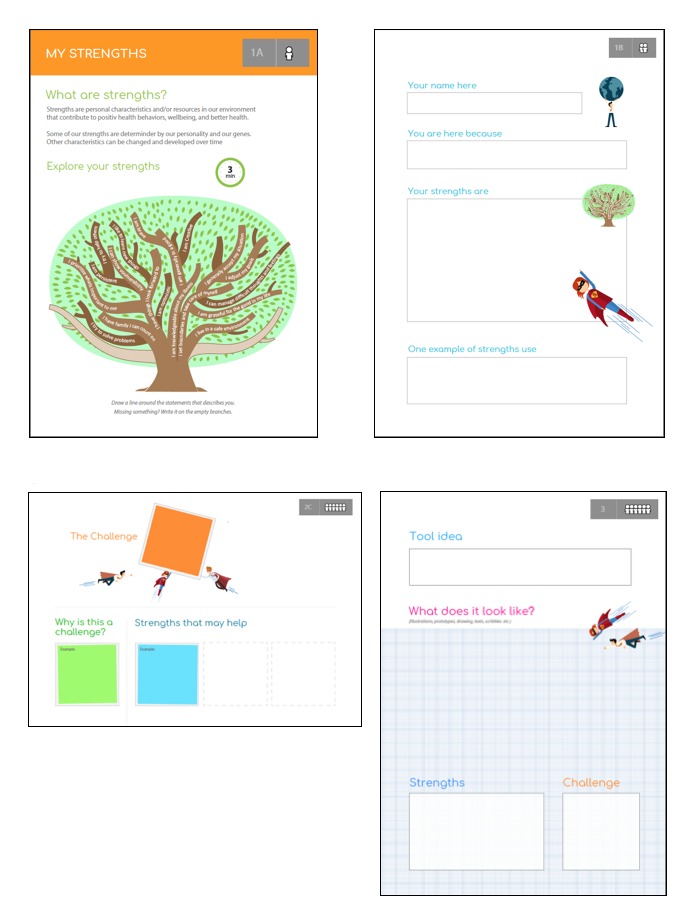
Poster templates: (top left) the strengths assessment and reflection exercise guide; (top right) the strengths interview guide and poster; (bottom left) poster for working and presenting challenge-reason-strengths connections; and (bottom right) poster for conceptualizing and presenting final group ideas.

The purpose of the first activity was to prompt participants to build positive, anticipatory images of themselves. In appreciative inquiry, this process is called establishing the “positive core”, and it plays a key role in changing people’s focus and collective attention to what is valuable, life giving, and vibrant in their life [36]. Therefore, in this task, each participant was asked to do a strengths assessment and reflection exercise. To support this process, each participant was provided a short premade text guide that showed examples of strengths that people with chronic illness from earlier studies had reported [13,39], and it visualized strength items as branches in a tree metaphor (Figure 1, top left). Participants were asked to select existing “strength branches” that describe their own strengths and/or use empty tree branches to write down new strengths items and describe one example from their life where they had used their strengths successfully. The goal of this exercise was to help participants articulate key strengths that they may wish to build upon in the future and to promote reflection of earlier situations in which they accomplished something positive by using these.

**Table 1:** Distribution of participants’ backgrounds across small groups.

Background	Group number					
	1	2	3	4	5	6
Patient (n=12)	1	1	1	3	4	2
Patient organization representative (n=3)	0	0	0	1	2	0
Health care provider (n=10)	2	3	1	1	1	2
Relative (n=2)	0	2	0	0	0	0
Information technology developer (n=4)	1	1	0	0	1	1
Designer (graphic designer, industrial designer; n=2)	0	0	1	1	0	0
Researcher (external, not part of the project team; n=2)	1	0	0	0	0	1

**Table 2:** Overview of data types gathered during the workshop.

Type of data	Description	Use
Audio Recordings	20:20 hours of plenary and group sessions	Primary data
Observation Logs	Reflection notes of facilitators and observers	Contextualizing
Photos	Photos of activities, ideas, scribblings, etc	Contextualizing
Written products	Sticky-notes, posters, note-papers, etc	Contextualizing

In the second part of the activity, the participants were asked to interview each other in pairs by following guidelines and fill-in fields of the premade poster template (Figure 1, top right). The poster was then presented by an interviewer to their small group. In addition to letting the participants become acquainted with each other, the purpose of these interviews was also to engage the participants to openly share stories about using their personal strengths and to inspire and energize the whole group with these positive stories.

#### Activity 2

In the second activity, all participants were asked to think about and report challenges and problems they perceived to be interfering with their overall well-being, as well as the reason why they were difficult to manage. While focusing on negative issues is a deviation from strict appreciative inquiry principles, recognizing challenges was an important part of making participants feel heard and acknowledged, especially patients who often faced substantial challenges in their everyday life. Therefore, we formulated this activity not so much as an activity that focused on the negative aspects, but rather as a first step in the process of changing the perspective and seeing the same challenges in a different light, which is also the main philosophy of a strengths-based approach.

After presenting the challenges and their underlying reasons on sticky-notes gathered on a wall, all participants voted for one that they would like to continue working on as a group. They were then asked to reflect and write down personal strengths that they themselves could use to overcome the chosen challenge. The reported strengths were used in a plenary discussion to expand the challenge-reason dyads into the challenge-reason-strengths connections (poster template presented on Figure 1, bottom left). The main reason for this step of the workshop was that strengths are often reported as highly context-sensitive [40,41], so enabling participants to report and see their strengths and challenges in an interconnected manner was considered an important part of the process. Additionally, this last step was designed to turn participants’ attention back to the “positive core” and in this manner, to set the stage for thinking about and developing new ideas for a future system that builds upon this concept.

#### Activity 3

For activity 3, the overall goal was to develop and conceptualize ideas for tools that could be used to support its users in identifying and using their strengths to address the selected challenges. The activity was organized based on standard guidelines for setting up codesigning activities and exercises and collaborative prototyping [35,42]. The participants were encouraged to brainstorm and create new and “crazy” ideas about possible tools. They were instructed not to think just about a technical tool but any kind of tool (eg, magical or fantasy solutions). The important part of this task was to describe how and why the tool could be useful and helpful. The last part of this activity was used to concretize the idea and describe the main features of the proposed concept by filling in a templated poster presenting their idea (Figure 1, bottom right).

#### Activity 4

Finally, each group presented their final ideas in a plenary session. After this presentation, all participants voted on the best idea and each group was rewarded with a prize in various categories. In addition to the idea with the most participants votes (peoples’ favorite), prizes were also awarded for most creative idea, best strengths collector, most inspiring idea, best idea for bridging people, and most magical idea. At the end, each participant was handed a diploma and a symbolic prize of a chocolate gold-medal.

### Data and Analysis

The workshop yielded multiple types of data, which are presented Table 2.

Two of the authors (JM, TK) made detailed summaries of all group discussions based on audio recordings and using observation logs, photos, and written products for contextualizing and gaining more details about the process. In addition, sections of audio recording relevant to overall workshop theme and specific activities’ topics were transcribed. The summaries and transcribed data were imported into NVIVO 11 (QSR International, London, United Kingdom) [43] for analysis. The data was categorized corresponding to the different topics that were addressed during the workshop (personal strengths, health challenges, creative ideas, and functionality and design requirements) and analysis was performed for each topic separately. Data were analyzed in several rounds by JM and TK according to the conventions of thematic analysis [44]. All results were discussed and inconsistencies, such as in understanding of design ideas, were jointly discussed until consensus was reached.

## Results

### Strengths

The participants reported a wide range of strength items during the first two activities of the workshop. In total, the participants contributed over 150 strength items. The reported strength items were sorted into six main categories or themes. The overview of the categories and example of strength items are presented in Table 3. The first four categories—my characteristics, coping strategies, resources in my environment, and behavior promoting health and positive emotions—relate to more general and personal strengths in people with chronic illness. In addition, we separated those strengths identified by health care providers and relatives that particularly pertained to their specific role.

### Challenges

In total, the participants reported over 100 challenges, which are summarized in the categories and examples shown in Table 4. Some of the challenges were related to performing general illness-related self-management tasks, such as understanding and managing illness and symptoms, communication with health care professionals, and gaining knowledge about the illness. However, a number of challenges were also related to living well, for example, managing and balancing everyday activities, establishing and preserving social relations, challenges at work and school, working on self-improvement, and gaining and preserving healthy lifestyle.

The challenges selected within each of the six groups are presented in Table 5, together with examples of strengths items participants proposed for addressing the selected challenges.

### Ideas

With the selected challenges as a point of departure, all groups managed to create ideas for tools or services that could help them to master the selected challenges while keeping a positive and constructive approach and promoting the utilization and mobilization of a person’s personal strengths during this process. To support the participants in out-of-the-box thinking, we, as mentioned above, gave no restriction for the scope of their ideas and proposals. The final groups’ ideas are described in Table 6.

### Functionality Requirements

During the group work, various functionality requirements were raised and discussed among participants. Main themes and examples of user requirements related to functionalities of strengths-based self-management tools are summarized in Table 7.

**Table 3:** Categories of strengths and examples of strengths items reported by participants in the study.

Categories	Examples of strengths
My characteristics (*I am*)	Solution oriented Goal oriented Like to learn new things Open for new possibilities Have a sense of humor Empathic Brave Able to show vulnerability
Coping strategies (*What I do/use*)	Set up clear goals Knowing my goals Being able to think and be positive Making plans Being aware of negative thoughts Knowing and setting my own limits Visualize things Stress management strategies Thinking long-term
Resources in my environment (*What I have*)	Support from family/friends A good health care system Support from health care professionals Support from peers
Behavior promoting health and positive emotions	Eating healthy Being physically active Finding and doing activities that give me positive energy
Specific for health care professionals	Good in communicating with people Focus on patient security Share information on different communication channels
Specific for relatives	Having knowledge about my rights as a relative Are involved in care processes at the hospital

**Table 4:** Categories and examples of challenges reported by participants in the study.

Main category of challenges	Example of challenges
Balancing everyday life and activities	Prioritizing important things Finding enough time for doing what you like Finding balance Managing time Finishing projects
Finding new ways to live with illness (mastering strategies)	Managing symptoms Spending time with family and friends Managing things as before illness Building better understanding of illness Learning how to adjust to environmental factors Accepting your situation
Challenges for being social	Socializing with other people Meeting new people Building relationships with other people
Work and school	Being at work or school Finishing school in time Limiting work load Keep motivation at work
Self-improvement	Being yourself Control thoughts and feelings Speak up when things become difficult
Relationship with health care providers	Communication between patient and health care provider Communication between relative and health care provider Access to knowledgeable people Health care provider does not see the whole picture
Getting new knowledge	Getting access to updated new knowledge Understanding complicated language
Healthy lifestyle	Doing physical activity Eating healthy Get enough sleep and rest Lose weight

**Table 5:** Challenges chosen by groups and strengths participants reported that can be used to address those challenges.

Chosen challenge	Examples of related strength items
Prioritizing important things	Willpower Able to ask for help and support Focused Prioritize my needs Solution oriented Optimistic Able to set and adjust my goals
Difficult to be a young adult in a hospital	Support from peers Independent Good communication skills Being able to think and be positive Well-functioning health care system open for improvements Have clear goals Will to learn new things
Finishing projects	Set up clear goals Taking small steps toward goal Enthusiastic Persevering Systematic Flexible to adjusting goals
Communication among relatives and the health care system	Having knowledge about my rights as a relative Brave, not afraid to say what I mean Creative Good and clear communication with health care providers Empathic Good network
Finding balance in life	Able to set my own limits Acceptance Knowledge about illness Prioritize things that are important to me Able to set and adjust my goals Good network Being able to think and be positive
Mastering various aspects of life	Grateful Goal oriented Plan the day ahead Stubborn Prioritize my needs Support from family/friends Being able to think and be positive

**Table 6:** Ideas developed during the group work and their short description.

App idea	Challenge	Short description of idea
Strengths treasure chest	Finding balance in life	A treasure chest app where a person can store his/her strengths, both those written by himself/herself and strengths added by others (eg, friends and family). The strengths can also be linked to different areas in life, and the app helps a person to plan how to use them, provides reminders about his/her strengths, and enables sharing information about his/her strengths with others.
Cheering squad app	Mastering various aspects of life	An app where a person can invite people to join his/her own cheering squad. People in the cheering squad can provide their personal support to the user by sending positive and encouraging messages through the app. The user has full control and can decide who he/she wants to invite and sets his/her own rules for communication (eg, no asking “how are you doing” questions, no need to respond and send answers to cheers).
User-controlled personalized hospital	Difficult to be young adult in a hospital	An app for a person transitioning from a pediatric ward to a unit for adults in a hospital. It provides support for establishing better and more clear communication with different people in person’s surroundings (eg, family, doctors, nurses). Some features include: always available and open communication channel with all parties, option for defining and sharing with everybody personal requirements and preferences, and link to a patient journal and information bank.
Empathy Simulator	Communication among relatives and the health care system	Virtual Reality 4D glasses that simulate experiences from different parties present in a consultation setting (eg, patient, caregiver, or a family member). The goal is to help a person experience the same situation from another person’s perspective. The glasses simulate what the person hears and sees and also what is felt and sensed.
Prioritizing app	Prioritizing important things	An app to help a person to make choices based on previous knowledge and experiences. The app supports a user during the process of making a choice—making a pro and con list for each option, help and tips on the strengths and resources one can use when making specific choice (based on previous experiences), and registering your satisfaction and experience with the selected choices afterwards.
Task-completer app	Finishing projects	A personalized app that helps a person identify his/her strengths and use them to complete challenges and tasks in everyday life. For example, it integrates sensors to understand when a person is stressed, and then prompts him/her with some of his/her strengths to help and motivate him/her to finish started tasks.

**Table 7:** Functionality requirements participants reported for strengths-based self-management tools.

Theme	Example of functionality requirements
Social support	Receiving support from others over, for example, online chat, calls, or messaging system
	Supporting others by for example sharing your knowledge and experience
	Connecting to other people (eg, peers, role models)
Support for patient-health care providers collaboration	Support for sharing information (symptoms, strengths, resources, and values)
	Supporting communication and collaboration
Awareness and reflection	Adding information about you, what is important to you, and current situation in different areas of life
	Overview of previous actions, goals, choices, and progress
	Identifying and providing overview of your current and previous strengths
	Registration and overview of how you use your strengths
Support for coping strategies	Support for prioritizing and making choices
	Exercises for identifying your strengths
	Exercises for mobilizing and building upon your strengths
	Goals (defining goals, defining and making small steps towards goals, support for achieving goal)

The most prevalent requirement across the groups was that technology should be designed to provide social support and connectedness with others. To accomplish this, features such as messaging, chat, or call were proposed as modes through which others (eg, friends, family, and peers) can provide support. For example, others could provide help during the process of identifying and reflecting on user´s personal strengths, or friends and peers can recognize user’s efforts, cheer him/her up, and support him/her for using and mobilizing his/her strengths in everyday life by sending positive and encouraging messages. In addition, the important part was also enabling the user to provide support to others, for example, by sharing knowledge and experiences in the common online space.

The other requirement often raised was supporting consultation with health care providers. In this context, participants proposed that technology could be used both in preparation for and during clinical consultation to facilitate sharing information between different parties (eg, patient, health care provider, or relative). Here, the main goal of the technology would be to facilitate a better understanding of each other’s needs, priorities, and limitations, and promote collaboration and cooperation supporting a person to see the situation from the different perspectives.

Other proposed functionalities centered on helping people to gain and further develop skills for raising awareness and reflection about themselves and their current situation. For example, this included implementing features for increasing awareness and reflection about personal characteristics and values (including strengths that one has), as well as monitoring progress and changes in different areas of life. The main goal of these features was to support the user in a process of reflection and learning about himself/herself and his/her current situation, goals, choices, and priorities, and make this knowledge available and capable of being reused in future similar situations.

Additionally, it was suggested that the technology should support people in transitioning from raising awareness to making concrete actions and plans, and developing coping strategies that could help them master everyday challenges. This could be done, for example, by helping them in prioritizing and making new choices and in defining the goals and small steps to reach these goals. An important requirement involved enabling and helping users to integrate their personal strengths, values, preferences, previous knowledge, and experience in this process, and to provide personally tailored guidance and support. Finally, adding support for exercises to help build and nourish personal strengths was seen as an important feature. Some proposed exercises were: registering good and positive experiences during the day, reflecting on positive experiences afterwards, and relaxation and mediation exercises.

### Design Requirements

In addition to functionality requirements, participants outlined various design requirements. Maintaining a positive focus was identified as an important requirement, especially in relation to giving positive and encouraging feedback to the user or framing the content in a positive way. Examples of how to make the design more engaging included using metaphors such as glasses, weight scale, tree, treasure chest, islands in the sea, and different worlds, or to use different visualization techniques such as dashboards, color coding of areas of life, smiley faces for registering daily mood, or the use of multimedia such as graphics and videos. Personal customization and adaptation to one’s own preferences and values were also raised as very important requirement both in relation to the user interface and features of the tools. Other user requirements included items such as giving the user full control, providing timely feedback, and making sure the tool was always available and accessible.

## Discussion

### Design Process: Combining Appreciative Inquiry, Participatory Design, and Service Design

In this study, we applied a new approach for involving stakeholders in the design process that builds on the core principles of participatory design and service design that emphasize enabling meaningful participation and collaboration with different stakeholders. Additionally, these principles were expanded with appreciative inquiry methodology that focus on integrating participants’ core values and on promoting positivity and creativity in the design process. This approach was also in line with the main principles of positive computing and positive technology, arguing that the design and development of technology should support psychological wellbeing and human potential, and foster a positive user experience. Combining principles from these different design approaches enabled us to create a positive and open environment that engaged and supported the participants to explore and propose new overall ideas, specific features, and contexts for the implementation of tools that expand users’ personal strengths and resources in a valuable and meaningful manner. For instance, the workshop started with an activity where the participants interviewed each other about their strengths, allowing them to both gain a better understanding of the concept of strengths by recognizing strengths in themselves and in others and to reflect and share positive personal experiences that they experienced while using them. Even though the literature reports that the concept of strengths can sometimes be both vague and difficult to grasp (especially when it is used in context of person’s health [45,46]), all participants in the study were able to identify and report their own strengths and integrate them into their groups’ work and ideas. Identifying and sharing their positive potentials also set the stage for a better common understanding, for collaboration in further group work, and for exploring the wider range of innovative ideas that build on these concepts.

Concluding the workshop, we gained overwhelming positive feedback from the participants, stating that the overall positive focus helped to keep them interested and engaged during the whole day workshop. This was also reflected through the amount and variety of participants’ feedback, as well as the themes and topics they discussed and the ideas that they proposed during the workshop. Thus, we can conclude that combining principles from participatory design, service design, and appreciative inquiry methodologies was an effective approach to enhance overall participant engagement, reflections, and creativity during the workshop with multiple stakeholders. This approach engaged them to think about new ideas and possibilities for technology that go beyond problem solving and addressing peoples’ deficits.

During the workshop, the varied backgrounds of participants contributed to producing innovative ideas that we, as researchers and designers, would not have considered alone without their insights. However, while stakeholders’ input about their challenges, preferences, needs, and values are crucial, creating successful new tools and systems also requires the specific knowledge and expertise of experts (eg, designers and researchers) to make sure the functional purpose, existing evidence, and the standards of good design are followed and addressed [42]. As presented in this study, a thorough analysis of the results of the workshop moved beyond the surface and concrete ideas. The inputs from the participants were also classified according to the types of functionality and requirements reported and the types of values and challenges these functionalities should promote and address. With this as a point of departure, the next step for researchers and designers is to integrate these inputs in the design of new and innovative tools and solutions that integrate user input and requirements and also build on theoretical and evidence-based frameworks and findings, such as the self-management models or the principles of positive computing or positive technology.

### Design of Self-Management Interventions

#### Concept of Personal Strengths

Assisted by the exercises and examples, the participants reported a large repertoire of strengths during the workshop, which ranged from personal qualities and characteristics (aligned with the character strengths within the field of positive psychology) to self-management and coping strategies (activities that promote positive emotions and healthy behavior) and supportive people and networks in their environment. This confirms previous research that shows that people perceive the concept of personal strengths in different ways and, when asked about their strengths, they report not just their internal personal characteristics but also external resources (eg, people from their environment) and positive and energizing behaviors that promote wellbeing and a healthy lifestyle (eg, knowing and working on the goals and healthy eating) [12,13,39].

This ambiguity of the term personal strengths reported by participants also influenced the idea-generating part of the workshop. While some of the groups stayed focused on a tool that could help people build awareness and mobilization of their personal strengths (*Strengths treasure chest* idea), other groups focused more on developing a tool that supported self-management activities (*Task-completer* idea) or social relatedness (*Cheering squad* app), where a component focusing more specifically on personal character traits was only a part of the app and not the main component. Having the groups initially either converging or diverging from the main concept of personal character strengths enabled further widening of the design space that afforded us rich insights into the variety of user needs and innovative ideas on how to use technology to facilitate integration of different types of strengths into self-management process.

#### Integrating Personal Strengths Into Self-Management Models and Interventions

Even though the concept of strengths was the main theme for this workshop, identifying participants’ challenges in daily life was also an important part of the process, both in relation to the design of the technology tools and its features. Such challenges constitute the larger context that tools for people with chronic illness should address. The diversity of challenges reported ranged from illness-specific challenges to more general everyday life challenges and are in line with previous literature [2,47].

Self-management programs and interventions (both online and offline) available today build on existing self-management models and are designed to address these known challenges. For instance, taking action is one of the main self-management skills defined in the self-management model by Lorig and Holman [3,48]. This includes developing mastering and coping mechanisms that support people in the process of changing their behavior (eg, making short-term action plans and performing them [49]). Other self-management skills are forming and keeping a well-functioning patient-provider partnership (defined in the same model by Lorig and Holman [3,48]) or building and utilizing better social support from family and peers [50]. However, as stated earlier, these strategies are most often implemented by emphasizing a person’s deficits and by focusing their attention mainly on a person’s problems and symptoms. Some examples of features implemented in technology-based self-management programs include: monitoring symptoms in an online symptom diary [51,52], teaching coping strategies to manage and lessen symptom burden [53,54], or providing training for improved collaboration between patient and care provider by facilitating joint discussions about symptoms and their management [55,56]. In line with a person-centered care approach, patients’ resources and strengths are starting to receive more attention in the field of self-management [57,58]. However, few technology-based tools that support patients in mobilizing their strengths are available today [39,59]. Therefore, the results of the present study provide valuable insights from stakeholders regarding the potential use of technology to integrate patients’ strengths as part of existing self-management programs, and to promote the process of creating better designed tools that also promote a person’s potentials, values and resources. Ideas from the present study such as the *Task completer* and *Prioritizing app* presents two concrete examples of how patients’ strengths could be integrated as part of self-management strategies for supporting patients to develop skills and take action. The app *Task completer* is not only helping the person to finish their tasks but also providing reminders to the user of his/her strengths to help him/her to remain motivated and to continue progress even in stressful situations. In the *Prioritizing app*, the system supports the user when making important choices and plans for the future by reminding him/her about his/her strengths, resources, and previous experience.

One other example proposed by the participants was how the patient-provider partnership and collaboration could be enhanced by integrating the topic of people’s strengths and resources in the conversation. In this context, it was proposed that technology could be used as a facilitator to support patients, relatives, and/or caregivers in consultation settings to identify, share, and jointly reflect on their strengths, resources, and values and how they could be used for planning more successful self-management activities and action plans (*Empathy Simulator* system). In this manner, the consultation could be enhanced beyond discussions revolving around symptoms and problems and provide support for more holistic person-centered care.

The findings from this study also support the importance of cultivating social relations, which in previous literature were identified as highly relevant ingredients of self-management programs [60,61]. Participants in this study recognized and reported the importance of people from their environment as a strength and proposed different approaches for how technology could further deepen relationships. All of the six tool ideas from the workshop contain at least some form of support for social interaction, including a shared platform for communication with different people from patient’s network, such as family, friends, doctors, and nurses (*User-controlled personalized hospital* idea), or creating a tool that promotes empathy and a common understanding between different parties (*Empathy Simulator* idea). Another example was the *Cheering squad* idea that included a one-way communication channel to the user, allowing others from the user’s network to cheer him/her on by sending supporting messages but not requiring the user to send feedback or a response. For chronic patients this is a very important feature since, due to their illness and severe symptom burden, this group often experiences a lack of energy and this is one of the main reasons that directly influences the erosion of contacts and network participation [62,63]. In this manner, designing social interactions can keep some of the motivational and engaging outcomes typical of social features [64] while also creating a safe and controlled space that does not require extra work from the user.

The described examples present just some examples and overall concepts that should be further explored and developed to accommodate the potential contexts where they will be used, integrate in more detail the stakeholders’ requirements and preferences, and build on knowledge from existing self-management models and interventions. The results of this study could be seen as an important initial step to guide the development of future research regarding the integration of personal strengths and resources in more advanced person-centered self-management programs and interventions.

### Designing for Quality of User Experience and for Promoting Human Potential and Wellbeing

The design of technology to promote a positive user experience and engagement is one of the main principles of positive technology (*hedonic level*) [26,27]. Previous related research proposed that this could be accomplished by: using virtual reality for fostering and manipulating joy and relaxation [65,66], using videogames and serious games for inducing positive emotional states [30], providing positive and encouraging feedback [67], and using engaging design principles (such as personalization, tailoring to ambient information, and applying metaphors in visual design) to provide a better user experience and to make the technology more appealing, engaging, and fun [31]. Positive user experiences and engagement were also raised as important requirements in this study. Participants proposed interaction- and interface-specific features, such as providing positive and encouraging messages and feedback to the user, framing the content in a positive way, and using engaging design features, such as metaphors or visualization techniques.

In addition to promoting a positive user experience, supporting self-actualizing and meaningful experiences that promote psychological wellbeing and human potential is one of the main principles of positive computing and an important pillar of positive technology (*eudemonic level*) [25-27]. Some previous research describing designing for meaningful and self-actualizing experiences include applying participatory design methods to explore how design could support autonomy, competence, and relatedness for young people living with asthma [68] or developing a mobile wellness-training app for people suffering from stress built upon the principles of acceptance and commitment therapy [69]. As the main theme of this study and the whole project was in line with this principle of supporting people in experiences that help them build on their potential, most of the ideas that participants proposed contributed to this overall goal. For example, the *Strengths treasure chest* idea that enabled users to easily add their personal strengths and receive reminders about them was suggested to provide boosts of positive emotions when the users were feeling down or under stress. Additionally, building upon and nourishing one’s own personal strengths and resources as part of self-management coping strategies (supported by the ideas behind the *Prioritizing app* and the *Task Completer* idea) can be seen as a way to promote both mastering of chronic conditions and also as a way to generate and build upon positive experiences and emotions for the user. Furthermore, the *Empathy Simulator* idea proposed a technology-mediated reflection of the experiences and sensations of others, promoting better understanding and empathy.

The ideas created during the workshop should be seen as overall concepts that have the potential to be further adapted and expanded, using principles of positive computing and positive technology to increase positive user experiences and user engagement [31]. Some approaches that are proposed in the literature that could be applied in this and/or similar studies are, for example, implementing a flexible design that adjusts to different situations and user characteristics and including social and cognitive prompts to keep people engaged with technology (eg, praise, rewards, or reminders) [31].

### Limitations

Although the number of participants was similar to the sample sizes reported in other related studies, the results were obtained as part of one workshop in one specific context. Additionally, the sample of participants was self-selected and some of the participants took part in earlier studies on personal strengths, which could have influenced participants’ engagement and interest with the theme of the workshop and the methods that we used. The great variation in the participants backgrounds could be seen as a limitation since the number of each type of participant per group was not large. Also, not all groups had equal distribution of participants with the same backgrounds which could potentially have influenced lower engagement of participants who were underrepresented in the group; however, on the other hand, this could have the positive effect of generating a wider variety of ideas that incorporate stronger viewpoints from various types of stakeholders.

Mixing participants in heterogenous groups during group work activities could potentially have introduced power disbalance in the groups and affect participants’ open participation and engagement during workshop. For example, a health care provider is usually seen by patients as having a power role, and as a result, patients may not feel as comfortable reporting dissatisfaction or frustration with their health care providers and whole health care systems in the presence of other health care providers [70]. However, if organized in the right manner, heterogenous groups can be highly effective and constructive by letting the participants exchange stories and build on different knowledge and needs stemming from their different backgrounds and experiences [71]. Therefore, to minimize the potential limitations of heterogenous group work in our study, different measures were applied: the rules of conduct that promote joined work and cooperation were clearly defined at the start of the workshop; the group work and activities was designed to promote participation, collaboration, and sharing ideas; and facilitators were trained to facilitate openness and active participation of all participants.

### Conclusions

In this study, we combined methods from participatory design, service design, and appreciative inquiry to jointly, with stakeholders, explore the requirements and codesign ideas regarding the use of technology as a facilitator for mobilizing the personal strengths required for overcoming the everyday challenges of people with chronical illness. Combining these approaches enabled us to create a positive and open environment where participants were encouraged and challenged to explore new ways of thinking that went beyond merely addressing the requirements for using a new technology. It enabled them to create new ideas regarding how technology can be designed to help them build on their values, potentials, and strengths, and it supported them in focusing more on these in everyday life.

The presented ideas and user requirements, combined with the insights from relevant theories (eg, positive psychology, self-management models) and the related literature, can be used to guide and inform the further development of self-management tools that promote a more person-centered strength-based approach. The findings can also be used as a starting point for additional designs of positive participatory workshops, which focus on identifying and co-creating ideas to develop and design future innovative strengths-based interventions, further promoting user engagement and positive experience.
